# Novel temperature responsive polymer based sealant for embolization

**DOI:** 10.1080/14686996.2024.2409059

**Published:** 2024-09-25

**Authors:** Ali E. Dabiri, Ravin Narain, Yi-Yang Peng, Wenda Wang, Max Itkins, Ghassan S. Kassab

**Affiliations:** a 3DTholdings, San Diego, CA, USA; b Department of Chemical and Materials Engineering, University of Alberta, Edmonton, AB, Canada; c Hospital of the University of Pennsylvania, Philadelphia, PA, USA; d California Medical Innovation Institute, San Diego, CA, USA

**Keywords:** Glue, atrial fibrillation, injectable hydrogel, self-healing hydrogel, lymphatic leakage

## Abstract

A sealant has been developed that improves upon current catheter-based treatments in the following ways: 1) Efficient delivery system, 2) No in situ polymerization, 3) No harmful byproducts, and 4) Cost-effective formulation. During the development process, particular attention was given to materials that were tunable, safe, and effective sealant agents. The thermo-responsive properties of poly(N-isopropylacrylamide) (PNIPAM) provides an ideal foundation to develop an optimized solution. Through a combination of model-based and material testing, a hydrogel was developed that balances conformational factors to achieve a customized transition temperature, radiopacity suitable for visualization, mechanical properties suitable for delivery via 3Fr catheter, sufficient cohesion once applied to resist migration under physiological pressures and an improved safety profile. Two applications, embolization of lymphatic leakage and exclusions of the left atrial appendage (LAA), to eliminate LAA dead space to reduce the risk of thromboembolic events, were considered. The material and benchtop results for this product demonstrate the suitability of this new material not only for these applications but also for other potential healthcare applications.

## IMPACT STATEMENT

A sealant has been developed that improves upon current catheter-based treatments in the following ways: 1) Efficient delivery system, 2) No in situ polymerization, 3) No harmful byproducts, and 4) Cost-effective formulation.

## GRAPHICAL ABSTRACT

**Figure:**
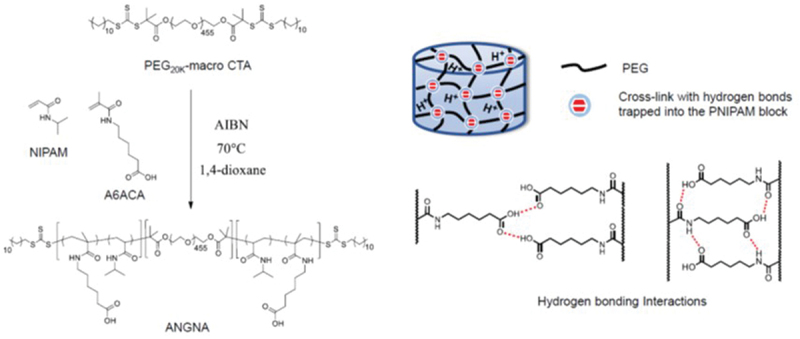

## 1. Introduction

Today, there are no clear best-in-class options for biomedical glue for clinical applications. Common materials are fibrin glue like BioGlue [1–4], Floseal Hemostatic Matrix topical [5], Onyx, and Trufill. The liquid-to-solid transitions of these materials make them particularly well suited to blocking small perforations and vessels where leaks are hard to localize. Their ability to rapidly apply in large volumes makes them a valuable solution for larger leaks with irregular morphologies. Despite their utility, most embolic agents are not radiopaque and require modifications to allow for precise application and fewer complications [6]. Trufill, a glue based on radiolucent cyanoacrylate, is often used with ethiodized oil to allow X-ray-guided embolization. Nevertheless, changes in the glue formulation to add radiopacity can also affect the cohesiveness and hardening timeline, demonstrating a material challenge for these technologies [7].

All catheter-based embolization agents have some disadvantages. Onyx, Trufill, Fibrin Glues, and others were all designed for vascular use. While off-label use is common, using a medical device in an anatomical region for which it was not designed can lead to poor results. For example, Onyx consists of dissolved synthetic polymer in DMSO. The polymer precipitates out of the solution as DMSO is carried away by circulating fluid [8]. This drastically delays the application time required for Onyx and causes a higher than intended DMSO concentration when it is used in the vascular system. As a result, it risks respiratory distress, pulmonary edema, vasospasm, and endothelial necrosis [8–12]. DMSO also poses a risk to patients with implants not tested for contraindication. These cyanoacrylate-based embolic agents have also caused neurotoxicity, hepatotoxicity, and edema due to formaldehyde release [13,14]. To avoid similar impacts on functionality and potentially harmful side effects, design criteria should be used from medical applications to inform the development of embolization technologies with better safety profiles.

Common embolization agents also have undesirable material properties. Sealants like Hystoacryl and TRUFILL**®** n-BCA rely on in situ polymerization and have an unpredictable polymerization window. This delayed chemical reaction can prevent the formation of a bolus of adequate volume, cause the spread of the sealant beyond the intended location, and adhere the catheter tip to the vessel wall [15,16]. Once in place, nBCAs have a strong adhesive effect but also cause vascular damage and inflammation. Although this enhances the embolization process, it raises questions as to whether the mechanism is valuable for long-term patient health. Further, even though n-BCA is used as a permanent embolic agent, embolization sites can re-open over time, potentially damaging vessel walls without the promised long-term treatment [17]. Onyx (EVA) has slightly more desirable material characteristics, offering customizable concentrations for release timelines and a formulation that precipitates over time to allow for more control over delivery. Unfortunately, it also has higher complication rates [18] Onyx has an 8.5% rate of catheter attachment to the vessel wall. Often, this results in the catheter being left inside the patient to avoid causing more damage [19]. As another example, fibrin glues can cause problematic secondary embolization and have shown only variable success as a lymphatic sealant [20–23]. This poor control over mechanical behavior illustrates two material challenges faced by these technologies: solidification timelines and controlled cohesiveness. Below, we will consider two specific applications of the proposed potential biomedical glue.

Atrial fibrillation (AF), the most common sustained arrhythmia, affects 3–6 million Americans and increases the risk of stroke by 4 to 6 times on average [24,25]. AF prevalence and disease burden increase with age, accounting for 15% of all strokes and greater associated morbidity and mortality than non-AF-related strokes [25]. In people >80 years old, AF is the direct cause of 1 in 4 strokes [24]. AF and related disorders have high individual and societal costs, ~ $26B per year in the US [26], and the incidence is projected to double by 2035 [27]. The left atrial appendage (LAA) is a windsock-like structure that extends from the LA (left atrium) and creates a side chamber, which can be a site of increased clot formation and accumulation in AF. The LAA in a low flow state, such as in AF, is the cause for more than 90% of thrombus formation [28,29], where the rapid contraction of the heart which accompanies AF can initiate the release of emboli and the consequent risk of stroke. Although the risk of thromboembolic events is reduced with long-term oral anticoagulation therapy, it is contraindicated in 7.8% of newly diagnosed AF patients [30], and only 50–60% of eligible patients with AF receive it [31]. Percutaneous [29,32–34] and surgical strategies [35,36] to exclude the LAA have been developed to reduce the risk of thromboembolic events while negating the need for anticoagulation and its related side effects. Current devices, however, have some complications (such as perforation and incomplete closure) and disadvantages (such as high cost and foreign body retention). Compared to endocardial exclusion, epicardial closure seems to have better hemodynamic and neurohormonal effects, but it is technically more difficult to achieve [34,37,38]. To overcome this pitfall, a novel concept has been proposed to achieve the elimination of the LAA dead space [39]. The endoluminal device and method include a simple intravenous approach followed by a trans-septal puncture where a suction device can be placed in the LAA, inverting LAA to the inside of the heart and holding it in place with tissue glue, i.e. no device left behind. The device and technique will eliminate LAA like the epicardial approach but from an endoluminal approach, which has never been done before [40]. The success of this approach is to access a suitable biomaterial glue that satisfies the design requirements.

The lymphatic system is responsible for recycling, immune, and waste functions in the body and hence it is critical to long-term homeostasis. Lymph node dissections, transplants, vessel reconstructions, and other surgical procedures may inadvertently damage lymphatic channels. Although less fluid flows through lymphatic vessels (several liters per day) than blood vessels, tissue, and organ injury can occur if left untreated [41]. Loss of fluid, triglyceride, lymphocyte, and immunoglobulin at leakage sites can lead to dehydration, nutritional deficiency, and immunologic dysfunction. Lymphatic leakage also increases susceptibility to infection, causing a cascade of harm if leaks go untreated [41]. Pain and prolonged hospital stay (about 12–20 days) are the most common effects of lymphatic leakage. Compression of vital structures can also occur in chylous ascites, lymphocele, and chylothorax due to increased tissue pressures. Lymphatic leakage treatment protocols are variable with delays in intervention ranging from several weeks to 2 months [42]. In some cases, 66% of patients in one study, fasting and medical treatment were sufficient to treat lymph disorders like small fistulas [43,44]. Conversely, some investigators believe early surgical intervention should be the first action for lymphatic leakage to control the leakage site, avoid complications, and shorten hospitalization [45]. Mortality rates for delayed treatment methods can reach 50%, while those for surgical intervention are only 10%. Surgical approaches include peritoneovenous shunt, lymphocytosis by suture ligation or embolization agent, and surgery combined with sealant [46]. While peritoneovenous shunt [47] and operation under direct vision [48] are the most common treatments, improved outcomes are driving the adoption of lymphatic embolization that is done with imaging, offering precision and fewer complications. If the leak can be identified, commercially available glues are used. There are several disadvantages to the current lymphatic embolization agents as they are not designed for the lymphatic system. Design criteria taken from the lymphatic system should be used to avoid impacts on functionality and potentially harmful side effects.

Here, we demonstrate a responsive sealant that leverages the customizable transition and mechanical properties of poly(N-isopropylacrylamide) (PNIPAM) hydrogels that addresses the disadvantages of the presently used glues. PNIPAM is perhaps the best-known thermo-responsive polymer, containing both hydrophilic and hydrophobic subgroups that facilitate a transition from water-soluble coil structures to water-insoluble globules with heating above a certain temperature. In a solution of water, that behavior enables viscosity and stiffness to increase substantially. Further, the initial viscosity and strength of the transition state can be engineered across a range of behaviors. Through co-polymerization with other monomers, we can tailor its thermo-responsive behavior and mechanical strength to create a hydrogel (liquid embolization material) that shape-fills upon injection at a wound site, adapting to irregular margins and sealing lymphatic leaks.

## 2. Methods

### 2.1. Preparation of Poly[(N-isopropylacrylamide)-co-(N-acryloyl 6-aminocaproic acid)]-b-poly (ethylene glycol)-b- Poly[(N-isopropylacrylamide)-co-(N-acryloyl 6-aminocaproic acid)] (ANGNA)

According to a previous report [49], reversible addition-fragmentation chain transfer (RAFT) polymerization was utilized to synthesize ANGNA. In brief, *N,N’*-azobis-isobutyronitrile (AIBN) (2 mg, 12 μmol) was first dissolved into 2 mL of 1,4-dioxane and subsequently mixed with 18 mL of 1,4-dioxane that already contained pre-dissolved NIPAM (1808 mg, 16 mmol), A6ACA (740 mg, 4 mmol) and CTA-PEG20K-CTA (1036 mg, 50 μmol). The mixture was degassed with nitrogen gas for half an hour and reacted at 70°C for 24 h. Termination of reaction was triggered by adding 5 ml of tetrahydrofuran (THF) into the reaction mixture. The polymer was purified by repeatedly dissolving in tetrahydrofuran and precipitating in diethyl ether three times. Last, the dried polymer was recovered after freeze drying.

All the materials used in polymer preparation were ordered from Sigma-Aldrich (Oakville, ON). *N-*acryloyl 6-aminocaproic acid (A6ACA) and macro-RAFT agent (CTA-PEG20k-CTA) were made according to the previous reports in the literature [50]. Hexane and toluene were used to recrystallize *N-*isopropyl acrylamide (NIPAM).

### 2.2. Hydrogel fabrication

ANGNA copolymer was dissolved into PBS buffer solution (pH 7.4) at 10 w/v% to prepare the hydrogels. The polymer solution was stored at 4°C for 24 h before use.

### 2.2. Characterization

#### 2.2.1. Catheter test

**Figure 1.**
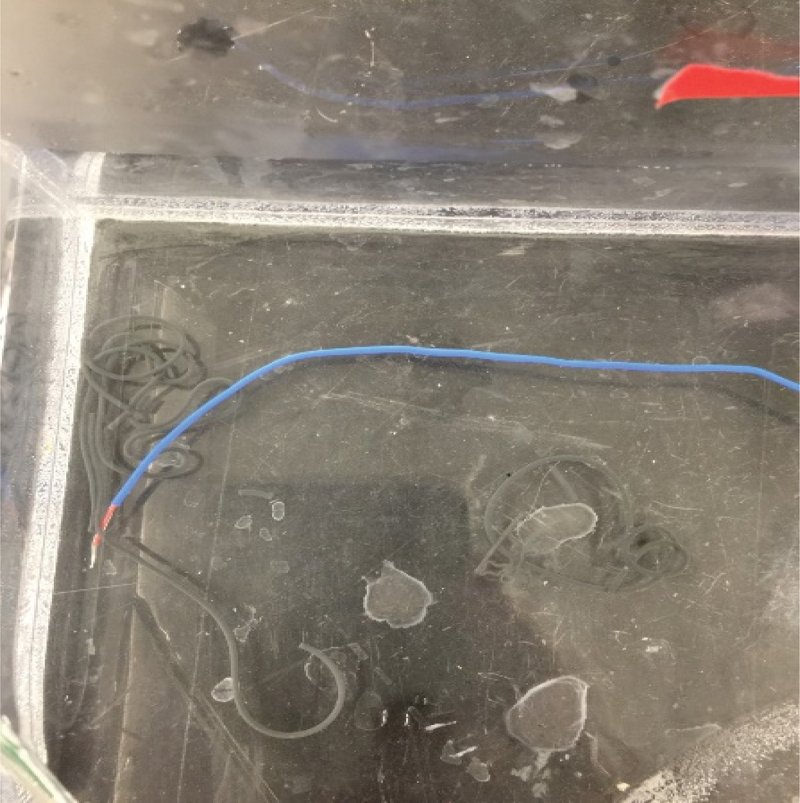
Hydrogel -tantalum mixture coming out of the catheter at 37°C shaped as noodles to demonstrate cohesivity of the glue.

A batch of glue was made using 10% v/w of glue dissolved in PBS having a pH of ~5. A test was then conducted with a 3Fr catheter. Saline solution was first tested on the catheter to ensure it was not blocked. To simulate in vivo conditions, 1 ml of the glue was tested with the 50 cm (out of a total of 90 cm) catheter placed in a water bath at 37°C. (We assumed that about half of the catheter is inside the body and the other half outside of the body at room temperature during patient operation). The test demonstrated that all the glue was transferred through the catheter easily. Tests were repeated several times. Hydrogel -tantalum mixture coming out of the catheter into the thermal bath at 37°C shaped as noodles to demonstrate cohesivity of the glue rather than being dissolved in the water as shown in Figure 1.

#### 2.2.2. Radiopacity test

**Figure 2.**
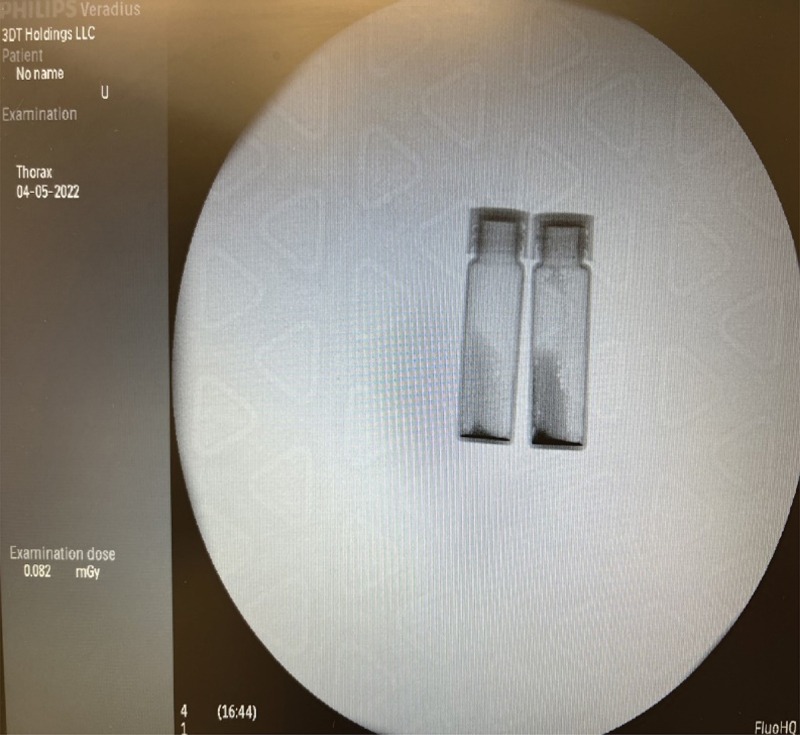
Radiopacity test, tantalum/glue ratio 1:4 (left),1:2 (right).

Two radiopacity levels (mass ratio of 1:2 and 1:4 tantalum to hydrogel) were made and compared against the other effects of tantalum in solution. Adding tantalum does not appreciably change the lower critical solution temperature (LCST). Hydrogel with tantalum with a mass ratio of 1:2 at room temperature was selected to provide higher resolution for enabling surgical visualization as shown in Figure 2. The catheter tests were repeated where the hydrogel was mixed with tantalum with a mass ratio of 1:2. Same results were obtained as before.

#### 2.2.3. Pull tests

**Figure 3.**
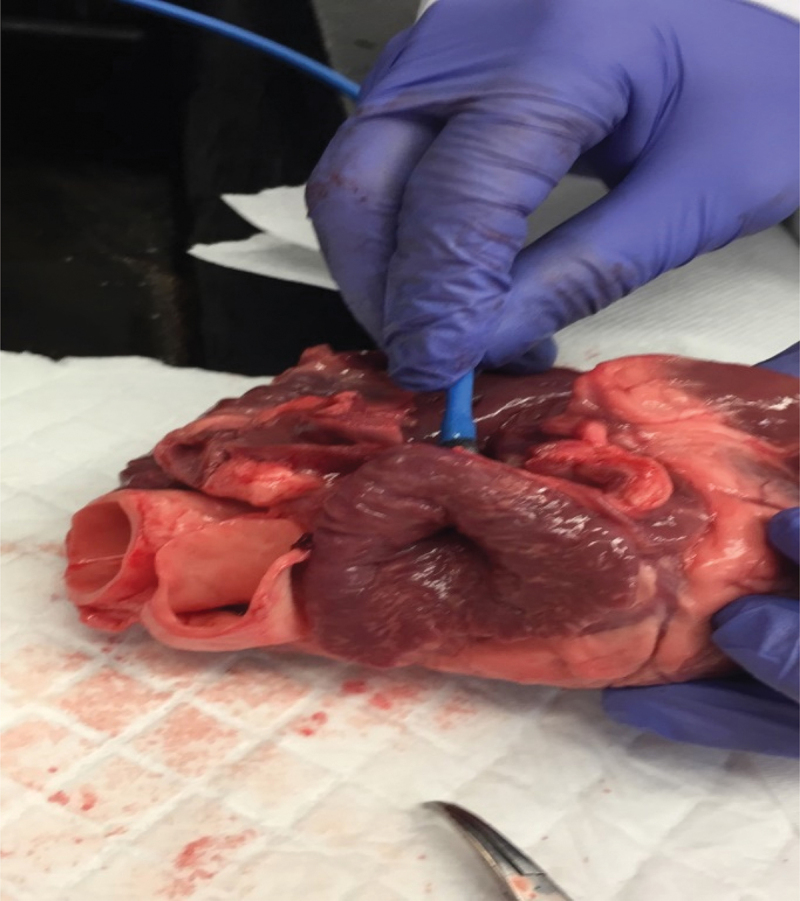
Inverted swine left atrial appendage to demonstrate the glue adhesivity.

**Figure 4.**
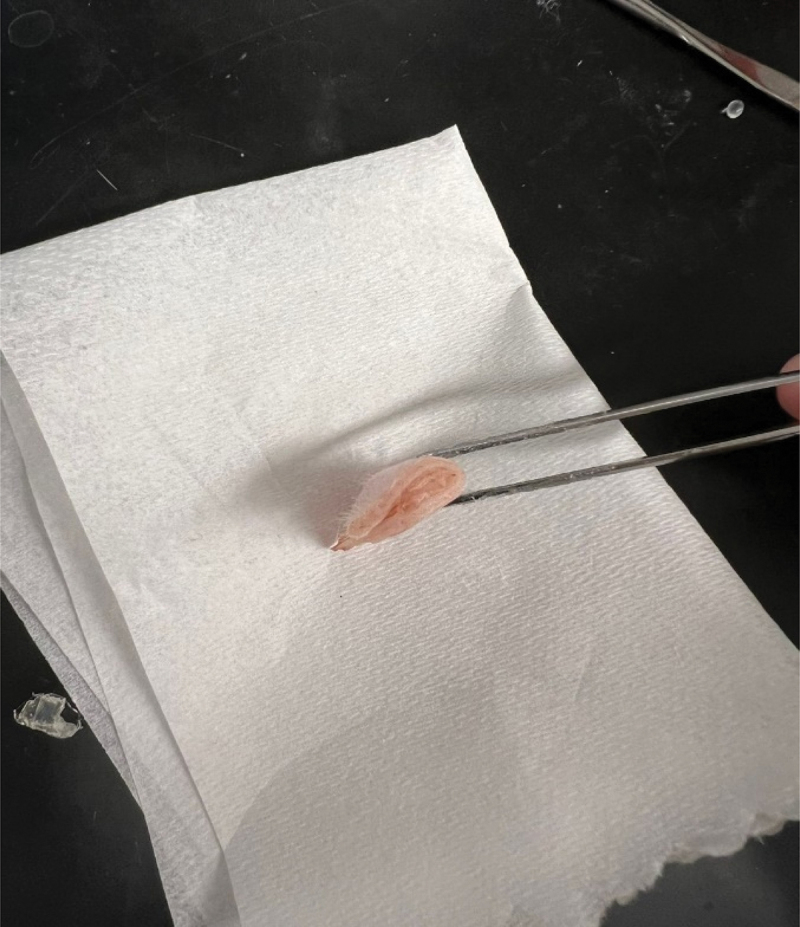
Fresh tissue glued together to demonstrate the glue adhesivity.

A swine heart was inverted as shown in Figure 3 and about 1 ml of crazy glue was applied and let dry for about 10 min. The inverted part was cut and placed under the pulling machine to measure the force required to separate the upper and lower portion of the inverted heart. The test was repeated three times, and the average force was about 3 N. The hydrogel was placed between two layers of fresh swine tissue at room temperature, as shown in Figure 4. It was then placed in the oven at 37°C for 10 min before cooling back to room temperature. The two layers of the issue were then subjected to a pulling test by a portable pulling device. The tests were repeated three times, and it took an average of 13 N before separating the two layers. The area under consideration was about 1.5 cm^2^. This force is considerably higher than needed to separate the inverted heart.

#### 2.2.4. Pressure tests

**Figure 5.**
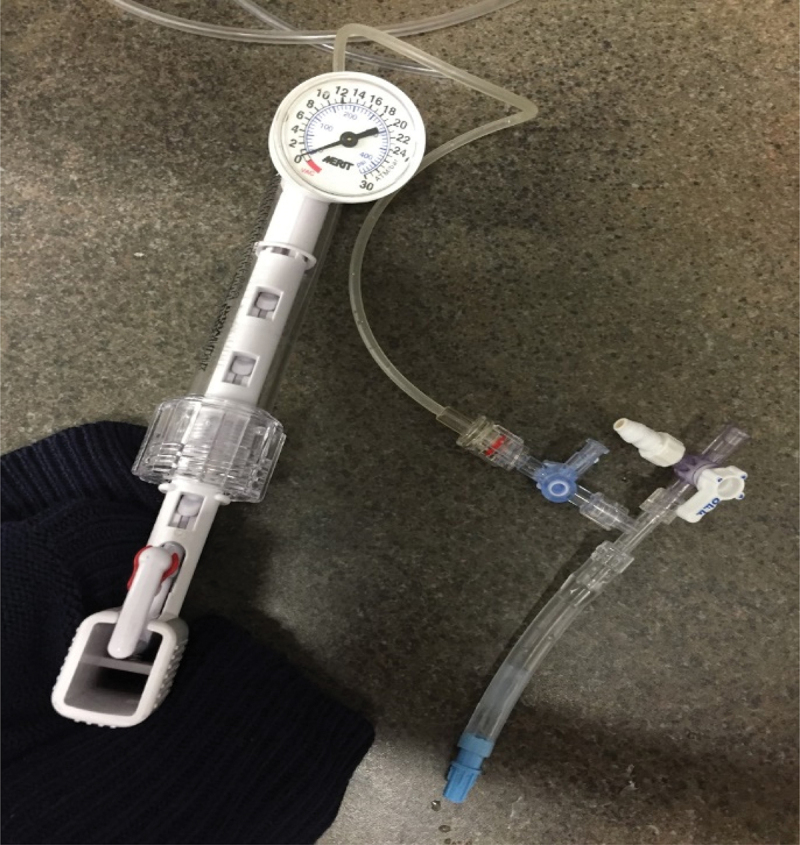
Pressure test to demonstrate movement of the glue against the applied pressure.

One ml of hydrogel was placed inside a 0.5 cm diameter plastic tube at 22°C. The plastic was inserted inside a 37°C thermal bath and waited for 10 min before applying 200 mmHg of air pressure, as shown in Figure 5. The hydrogel did not move under pressure at 37°C.

## 3. Results

We have demonstrated that the first generation of ANGNA characterization has met the expected outcome. It takes around 45 s to deliver the desired glue amount (1.2 ml is generally needed for these applications, which results in almost 1 ml of solid glue) through a 3Fr catheter, where we have reached our goal of less than 1 min with the hydrogel-imaging agent mixture. After injection, the ANGNA solution can rapidly transform into a stable hydrogel material and maintain the structural integrity in a water bath at body temperature due to the strong intermolecular interactions (e.g. hydrophobic interactions and hydrogen bonding interactions) between the polymer chains. The addition of tantalum enables the hydrogel to be visualized under X-ray, showing promise for interventional visualization. Moreover, the hydrogel also demonstrated excellent tissue adhesiveness. The pulling force to separate the glued tissue by using ANGNA hydrogel was 4 times higher than the crazy glue. Such high adhesion allows the hydrogel to tolerate a burst pressure as high as 200 mmHg, without detaching from the tissue surface.

## 4. Discussion

This work demonstrated that a NIPAM-based hydrogel has the potential for use in the exclusion of LAA or lymphatic leakage. By tailoring molecular structure, concentration, and additives, an appropriate prototype showed sufficient durability and cohesion to withstand a basic requirement.

Our goal was to overcome the mechanical, biocompatibility, and delivery shortcomings of existing technologies while meeting standard requirements for efficacy. The results presented here demonstrate a temperature-responsive sealant that can be injected as a low-viscosity liquid via a 3Fr catheter to a site of LAA or lymphatic leakage, and then undergo solidification to create a form-fitted, non-biodegradable embolization. In a clinical setting, this technology would provide interventionalists with a valuable alternative to existing embolic agents through safer materials that are easier to handle. The developed glue material is novel in that it 1) has a phase transition specifically tuned for delivery via catheter, 2) is pre-polymerized yet offers significant cohesion to exclude LAA or lymphatic leakage, 3) balances radiopacity with hydrophilicity to have an optimized solidification window, 4) does not introduce chemicals with harmful side effects into the body, 5) will significantly lower the price of treatments, and 6) will be the first sealant agent designed specifically with the LAAI or lymphatic leakage in mind. The results presented offer proof of concept and characterization intended to demonstrate the viability of this formulation.

**Figure 6.**
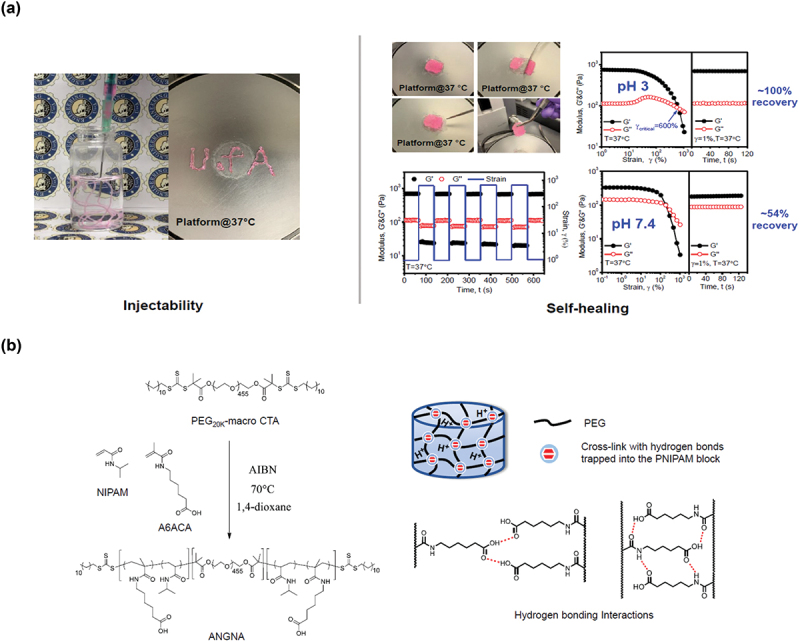
Temperature responsiveness and mechanical properties of ANGNA. Figure 6a (left) shows the injection of a 4°C-preserved 10 w/v% polymer solution into a phosphate buffer solution (pH 3) at 37°C and facilely writing letters ‘U of A’ on a platform at 37°C (the hydrogel was dyed with rhodamine b). Figure 6a (right) shows the thermosensitive storage (G′) and loss (G′′) modulus of a 10 w/v% hydrogel (Reproduced with permission from [51]). Figure 6b (left) shows the synthesis of the triblock copolymer using a PEG difunctional macroRAFT agent. Figure 6b (right) illustrates the possible mechanism of the hydrogel formation through hydrogen bonding interactions.

A biological environment adaptive supramolecular assembly strategy has been recently developed for the preparation of an injectable self-healing hydrogel with tissue adhesive property for the treatment of gastric perforation [51]. The hydrogel was made by a triblock copolymer, Poly[(*N*-isopropylacrylamide)-*co*-(*N*-acryloyl 6-aminocaproic acid)]-*b*-poly (ethylene glycol)-*b*-Poly[(*N*-isopropylacrylamide)-*co*-(*N*-acryloyl 6-aminocaproic acid)] (ANGNA) as shown in Figure 6. Figure 6a (left) shows the injection of a 4°C-preserved 10 w/v% polymer solution into a phosphate buffer solution (pH 3) at 37°C and facilely writing letters ‘U of A’ on a platform at 37°C (the hydrogel was dyed with Rhodamine B). Figure 6a (right) shows the thermosensitive storage (G′) and loss (G′′) modulus of a 10 w/v% hydrogel. Figure 6b (left) shows the synthesis of the triblock copolymer using a PEG difunctional macro RAFT agent. Figure 6b (right) illustrates the possible mechanism of the hydrogel formation through hydrogen bonding interactions. Increasing temperature to physiological temperature can trigger the gelation of the polymer solution due to the hydrophobic interactions between the dehydrated PNIPAM block. At low pH, the randomly incorporated A6ACA groups can form hydrogen bonding interactions with both face-on and interleaved configurations, providing improved material stability and self-healing capability of the hydrogel. The interfacial hydrogen bonding interactions formed between the A6ACA groups and the amine/carboxylate groups on the tissue further render the hydrogel with tissue adhesive property, and it was validated by demonstrating strong adhesion between the hydrogel and wet porcine stomach tissue. Furthermore, excellent antibiofouling property and biocompatibility were proved by conducting bacteria adhesion assay and MTT assay, respectively. These properties further reduce the concern of using this hydrogel as a glue in the biological system. The hydrogel material has been validated in *an in-vivo* animal model (rat) for the treatment of gastric perforation.

## 5. Conclusions and future studies

A simple, iterative design process was developed for a suitable prototype for a new biomedical sealant. While the work herein is promising, significant challenges remain in establishing efficacy and reliability compared to existing procedures. Consistent material properties across batches and long-term sealant stability remain undetermined, along with the many other forms of validation required for new medical devices. Nevertheless, this work demonstrates that promising alternatives to standards of care need to be further explored. Future work will be to develop the second-generation sealant, including chronic and acute disease swine testing and refining the sealant product. Significant testing will still be required to show long-term stability of the hydrogel, and reliable material properties. Additionally, monomer conversion ratios must be more closely examined to isolate a precise material specification. Should these various metrics be obtained with suitable results, it is anticipated that the technology will have many other biomedical applications that will be readily attainable with the map of chemical variables gained via this research. Future work on these technologies will also require examination of scalability and simplicity of the manufacturing process to result in a large batch approach, developing a commercialization plan, formal quality control with validation, GLP animal studies, and submission for an IDE first-in-man study.
